# Cases Illustrative of Bowel-Diseases Prevalent in the Crimean Expedition during the past Winter

**Published:** 1855-12

**Authors:** William Smart

**Affiliations:** Surgeon of the Naval Brigade, Balaclava.


### TRANSACTIONS

OF THE

### EPIDEMIOLOGICAL SOCIETY OF LONDON.

### Papers and Communications.

### CASES ILLUSTRATIVE OF BOWEL-DISEASES PREVALENT IN THE CRIMEAN EXPEDITION DURING THE PAST WINTER.

By William Smart, M.D., Surgeon of the Naval Brigade, Balaclava.
Communicated by Dr. McWilliam.

The two cases which are the subject of this paper, are good examples of diseases that have existed to a formidable extent among our forces; and, as I believe them to have arisen from causes as distinct as the symptoms during life and the lesions found after death have been different from each other, I shall endeavour to detail as fully as possible the peculiar characters of each.

The first case is nosologically arranged as "Dysentery"; it terminated by ulceration of that variety, I think, termed *pustular* by Blane and other authorities on the medical history of former campaigns. The second case is classed "Diarrhœa", the name of a symptom only, not even persistent in the course of the disease, and which cannot convey any idea of morbid conditions which terminate in general softening without ulceration of the villous layer of the mucous membrane of the intestines, and which arrest life by destroying that absorbing power by which the chylous products of digestion are introduced into the system.

I regard the cases about to be given as types of the diseases named dysentery and diarrhœa, both most fatally prevalent here during the past winter.

Case i. A petty officer of the *Queen* was brought to me from the camp on the 1st Feb., with the following report:—"Was admitted on the sick-list on the 16th December with diarrhœa, which has become a bad case of dysentery. Blue pill, ipecacuan, and opium have failed to relieve. The evacuations are more mucous than sanguineous." When received on board, he appeared suffering from the fatigue of the journey to Balaclava. He was not greatly emaciated. The muscular parts felt soft and flabby, and he stood up with difficulty from pains in the muscles of the thighs and legs. The surface was chilled and dry, and conveyed a harsh sensation to the hand. The face was pallid, and the eye pearly; the gums were injected; the tongue was coated by a thick yellowish-white fur at its base and centre. The voice was low, and he complained of thirst, of dryness in the pharynx, of tormina about the lower region of the abdomen, preceding the desire, and of tenesmus during the act of evacuation. The evacuations were frequent, eight to ten in twenty-four hours, sero-sanguineous, not fæculent. There was a very general sub-acute soreness of the abdomen on pressure, especially along the track of the large intestine. The pulse was 90, small and soft. The urine scanty. He had occasionally hiccough.

The most prominent features of the case were asthenia of the nervous and circulating systems; scorbutic signs in the soft solids; hæmorrhagic disposition in the mucous membrane of the bowels, and sub-acute inflammatory action of a low form affecting the large intestine.

*Feb.* 2. The evacuations were rather less frequent. *Feb.* 3. Constitutional powers unimproved. He took the recumbent position, as that on either side caused pain and brought on a desire of stool. His evacuations were observed to emit an offensive sickly odour, and to have undergone an alteration of character, being free from scybala and coagula, and containing a light-coloured flaky sediment, which subsided in a reddish watery fluid. *Feb.* 4. The hiccough increased in frequency. There was general soreness of the abdomen, and slight impressions of the fingers were left on it, as if from an œdematous condition, while deeper pressure over the colon imparted a slight gurgling sensation. The sphincter ani was relaxed, and there was an involuntary action, and, at times, the desire for action of the bowels was urgent. There was also some loss of power about the neck of the bladder, which produced frequent but painless micturition. *Feb.* 5. Complained of having no sound refreshing sleep. He lay in a state of slumber, sighing and moaning, at times unconsciously. The systemic powers were declining. He spoke in low tones. The pulse was 110, soft and undulating. The skin was always cool and dry, except over the abdomen, where its temperature was raised. There was noisy singultus with dysphagia from pain in the œsophagus, apparently from retroverted action, and this made him less willing to take food. The tenesmus was rarely felt, and there was constant draining away of discharges, which imparted a sickly odour to his bedding and person. *Feb.* 7. Singultus, dysphagia, and involuntary action of the bowels continued. He lay in a drowsy, half-conscious state, from which he was easily aroused, and at intervals he was restless. There was inflammatory redness over the sacrum, and the anus was excoriated. *Feb.* 8. Singultus and tenesmus had ceased; he avoided the effort of swallowing; the urine was scanty; the surface was chilled; pulse, 140 and thread-like. He lay in a state of slumbering quiescence. *Feb.* 9. He lay tranquilly all the day, offering no complaints, nor expressing any wants or wishes. The energy of the nervous system appeared to have been entirely exhausted by a draining, irritative disease, and by want of continued sleep. He expired at 5 P.M.

The principles of treatment adhered to were, to moderate the inflammatory action by fomentations and blisters over the abdomen and seats of pain; to impart tone to the lower bowel by mildly astringent sedative injections; to improve the vitiated secretions of the mucous membrane, liver, and pancreas by gentle alteratives, combined with the anodynes and antispasmodics; and to nourish the body and support the vital powers by frequently administering a mild unirritating diet, and latterly, by the careful exhibition of wines.

*Autopsy, seventeen hours after death.*—A considerable amount of adipose tissue remained on the trunk and limbs. The omentum was thin and shrunken; the mesentery had a good quantity of fat; no fluid in the peritoneal sac. The external surface of the large intestines and of the ileum presented a livid vascular appearance. In removing the intestines, they were torn across at the descending colon, owing to the extensive ulcerations of the mucous membrane; the internal surface, corresponding with the external discoloured portion, was studded with ulcerations, in many places so thickly set as to resemble the ulcers of confluent small-pox. These ulcers extended from the lower part of the rectum to a foot above the ileo-colic valves; they were very closely set as high up as the cæcum, where they become more scattered, but at the ileo-colic valves they were again confluent, and these valves were much thickened by interstitial deposit in the sub-mucous tissue. Ulcers of smaller size, gradually decreasing in number, were found as high as a foot above the ileo-colic valves, and, as far as they extended, the mucous membrane was highly vascular and much softened, and had the valvulæ conniventes effaced. The liver presented no remarkable condition, except an easily broken tissue posteriorly, where the systemic vessels make their entrance and exit. The gall-bladder was moderately full, and the liver in its vicinity was tinged with bile.

*Remarks.*—This man's disease did not make the rapid progress of acute inflammation; he had been under treatment in the camp since the 16th December, and, according to his own statement, he had suffered more or less from looseness of the bowels from the time of his debarcation in October. The minor degree of emaciation he had undergone showed that the nutritive functions had not been greatly impeded. There was no proof of a condition of the system approaching to fever having at any time existed. Certainly, while on board, he had none of the signs of fever, not even of a low or hectic type, such as are looked for where destructive inflammatory action is progressing to a fatal termination by ulceration.

The want of nervous power was evident in the broken rest, the somnolent condition, and, at a later period, in the loss of control over the sphincter ani. And this exhaustion of the nervous energy, inducing a diminution of the tone of the circulating system, that adynamic condition was induced which is best displayed in the tendency to slough in those parts endowed with lowest vitality. Together with these alterations, the blood appeared to have undergone a change of its crasis, which admitted of its transudation in a sero-sanguineous form from the mucous surface, the seat of the diseased action, the secretions of which were also depraved. That the whole system was under the influence of a strongly scorbutic taint, appeared from the loss of cohesion and the vascular state of the gums, the peculiar debility of the limbs, and the great depression of the animal spirits.

In the sequence of morbid changes it would seem that disorder of the secretions of the intestinal canal and of the viscera that subserve to the functions of the *primæ viæ* preceded; and, that when the causes which produced the scorbutic cachexy had lowered the vital energies, the disorder of the bowels assumed an aggravated form. Then the diarrhœa became converted into dysentery, and that disease (of which the natural tendency, when implanted on adynamic states of the system, is to lesions of structure, resting on a diseased basis,) produced rapid disorganisation of the structures in which its pathognomonic actions are developed.

The evacuations were truly indicative of the disease. After the morbid lesion had passed into an ulcerative stage, the mucous or bloody fluid of dysentery was not seen, and the evacuations might be easily regarded as those of chronic diarrhœa, rather than of dysentery in any form. They were copious, but rarely of one consistence; a watery fluid tinged with blood or bile had in it a subsidence of a flocculent or grounds-like form, and of a dun or light yellowish colour; their odour was peculiarly offensive. Scybala are not uncommon in those evacuations, but I have never seen coagula or streaks of blood in them; the blood that was in them appearing to be poured out by exudation, and therefore incoagulable.

Case ii. A seaman, 22, belonging to the *London*, was received from the camp on the 1st Feb., where he had been under treatment for diarrhœa since the 22nd of January. He stated that he had suffered repeated attacks of it previous to that date. He was landed on the 2nd of October. The medical report received with him was as follows: "Diarrhœa; he is much emaciated, evacuations about two in a day, but he has lost strength so much that his case demands hospital treatment."

From this it would appear that he was sent away, not on account of the immediate urgency of the diarrhœa, but on account of the *marasmus* that had supervened.

On his reception on board he was found to be greatly emaciated, and extremely reduced in strength, but he still retained a certain cheerfulness of manner, and his mind was clear and intelligent. The surface was sunken, cold, and harshly dry; eyes sunken, and having but little secretion; gums pale and bloodless; tongue broad, pale and flaccid, having no erect papillæ. The pulse was 110, and very feeble, thread-like. The urine of average quantity. He made no complaint of pain on pressure of the abdomen. The first evacuation on board was thin, yellowish, and fluid, but not serous or watery. The physiognomy and symptoms were much like those common to the diarrhœa of cholera epidemics. *Feb.* 2. Slight reaction. Pulse 90, with great force. Three evacuations in twenty-four hours. He took food well, and there was no marked tenderness of the abdomen on pressure. *Feb.* 3. Purging rather more frequent. Stools light-coloured, semi-fluid, and of fæcal odour. The tongue was pale, dry, and glazed. Pulse 110, weak, and more wiry. He desired food, and he had control of the sphincters. *Feb.* 4. In the same state. The tongue resembled the surface of parchment. The urine was secreted in average quantity. The conjuctva bore a slight mucous secretion. The abdomen bore pressure well. Stools frequent. There was a marked alteration for the worse. Diarrhœa of light-coloured stools continues. He had passed from a state of extreme depression to one of irritative action based on prostration. The secretions from the lachrymal and salivary glands had ceased. The abdomen bore pressure well, and this moderated alarm as to his condition, but he emaciated rapidly, although taking a fair amount of nourishment. *Feb.* 5. He sank in his bed, and lay with the lips and eyelids half opened, and with the teeth and eyeballs exposed and dry. He was in a state resembling syncope, from which he started on being spoken to. The diarrhœa caused no distress. Pulse 110, and extremely feeble. *Feb.* 6. He was more somnolent. Eye pale and secretionless, the pupil natural in size, and active in its contractions. Urine natural. The diarrhœa was checked in frequency. *Feb.* 7. The evacuations were of the same character. He was quite rational and quick of apprehension when aroused from his somnolent condition. He made no complaints of pain. *Feb.* 8. No diarrhœa since the 6th, and the abdomen bore pressure well. The nervous force was being somewhat recruited, and the signs of sinking seem to arise from faults of the vascular system. Tongue moist. Asked for food. *Feb.* 9. An evacuation of the same character as before. Although much care had been taken to protect him from cold and variations of temperature, there was found to be a gangrenous spot on the left little toe, and most of the toes of the left foot were in a doubtful condition. *Feb.* 10. He made no complaints, took food, and asked for wine. No return of diarrhœa. *Feb.* 11. Pulse imperceptible at the wrist. He fell back in a state of syncope from the half sitting posture in bed. No desire for food. Evacuations of same character. Urine limpid, amber coloured, of normal quantity. *Feb.* 12. Tongue dry and bloodless. The toes of the left foot were insensible, and they had darkened grey spots, as if from "dry gangrene". The lower limbus of the corneæ were leucomatous from continued dryness of the part, and the absence of interstitial exhalation. *Feb.* 13. He laid tranquilly, made no complaint, expressed no wishes. The "leucoma increases." No pulse at ankles or wrists. The gangrene was confined to the toes. The mind was intelligent. The voice was whispering. He seemed to pass urine freely. A stool passed unaltered from former characters. *Feb.* 14. Gradually sinking. At noon, when incapable of sensation in the feet, and unable to see from the opacity of the cornea, he expressed a wish to smoke a little tobacco. At 7½, P.M. Life departed from anæmia, the result of inanition.

The main indications of treatment seemed to be to put an end to the diarrhœa, which had induced exhaustion. And an attempt was made to effect this by means of small doses of calomel with camphor, and a moderate allowance of wine; and when some reaction had occurred, by chalk and opium. On the recurrence of symptoms of increasing exhaustion, every effort was made to support the system by beef-tea, arrow-root, and port wine; and in a later stage by ammonia and mixture of spirits of wine. By external warmth, frictions, and stimulating embrocations, care was devoted to maintaining as far as possible, the feeble circulation in the extremities.

*Autopsy fourteen hours after death.*—The body was extremely emaciated; but there were no gangrenous spots on the sacrum or trochanters. There were dark gangrenous spots on the toes of the left foot, and the small toe had separated at the first phalanx. All the fat had been absorbed, and the areolar tissue was dense, and was not anywhere infiltrated by fluids. The muscles had a healthy red colour.

*Cavity of the Thorax.*—Lungs grey and perfectly healthy. The heart was small, and had a fringe-like body on the exocardial surface over the left ventricle towards its apex. No fluid in the sac, and no corresponding condition of the opposed pericardial lining. The cavities and valves were normal. The large arteries were quite empty; the great veins contained but little blood.

*Cavity of the Abdomen.*—No fluid in the peritoneal sac; the omentum was thin and shrunken; the walls of the intestines were thin and semitransparent where inflated; the mucous surface was of a fawn colour, and had frequent venous-coloured, arborescent patches of vascularity; it was softened throughout the whole extent of the intestinal canal, and was everywhere easily removed. In the large intestine there were a few spots where the villous surface had been removed, apparently by pressure of the fingers in excising the intestines; certainly these excavations did not bear the character of ulcerations. Chyme was found in the stomach and upper intestine, and in the lower intestine there was fluid fæcal matter of the same character as that evacuated during life. The mesentery was very dense in its areolar tissue; its veins were congested; the mesenteric glands were not enlarged; and the lacteals were not apparent. The thoracic duct, traced through the mediastinum, was found to be empty. The liver was large, and resisted on incision, and the cut surface was paler than natural. The gall-bladder was distended with healthy bile. The pancreas was large, and very firm in tissue. The spleen was rather small, also firm. The kidneys were without signs of discase, and the bladder contained limpid urine.

The patient had suffered attacks of diarrhœa previous to this, while serving ashore, but this last attack was early attended by emaciation. There was no evidence of fever previous to his removal from the camp; and on board this ship his condition never exceeded that known as "irritation".

Pain on pressure of the abdomen, which, as a symptom, is an ordinary criterion in bowel diseases, was not, at any time, of a degree to arouse suspicion of inflammatory action.

The character of the evacuations was always peculiar and unvarying, and it may said, in a great measure, indicative of the disease; the appetite not being absent, and the functions of the stomach being performed, and there being no actual proof of disordered hepatic function; the alvine evacuations had a frequency proportionate to the quantity of food taken, they were of a semi-fluid consistence, not watery, of fæcal odour, and of light colour from the excretion, most probably, of the chyle-globules, which the diseased villous surface of the intestines had failed to absorb.

It would appear that in cases of this character, while the lacteal absorbent system is entirely unemployed from lesion of the absorbent villous surface, the lymph absorbents that are distributed to the tissues of the part are equally active with those of other parts of the body, because it is seen that emaciation goes on in them in equal degree; for the coats of the intestines become almost diaphanous; the adipose tissue of the omentum and mesentery is absorbed, and those membraneous parts are reduced to their mere basement of areolar tissue. The tongue assumes a peculiar appearance, which may be relied on; it is pale and broad, lax in its fibre, and has flaccid papillæ; it is rarely coated or foul, and, as disease advances, it becomes dry, and has a glazed parchment-like surface. The body wastes rapidly, for the reason that no new material is added to supply the expense of tissues; but, though this wasting is remarkable, the tissues, excepting the mucous membrane—the seat of actual disease—are all increased in firmness; and the disposition, in parts endowed with low vitality, to slough when subjected to pressure, does not exist. The blood, constantly diminished by nutrition and by excretion, is not replenished by the sanguifying organs. It ceases to supply material for the less important secretions, such as the tears and saliva. It ceases to supply the water for perspiration, and, towards the end, the urine is diminished in quantity, and may even be suppressed. The force of the circulation is constantly being reduced, until from the inability of the heart to propel its blood, syncope will occur in the half sitting posture in bed; the pulse is not felt at the ancle and wrist; and, if the patient is submitted to a lowered temperature, the extreme points fall into a gangrenous condition, resembling that of old men more than the sphacelus of frost-bite. The sensorium does not greatly resent the changes that are making fatal inroads. In the early stage, the mind is usually clear and intelligent, and it remains coherent to the last. Sensations are received and volitions conveyed with normal force; and when, from anæmia of the brain, or from deficient force of the heart to propel a sufficient column of blood to the brain, there is a tendency to syncope and somnolency, that condition differs greatly from the coma of fever and from the insensibility present in compression of the brain, as the patient is easily aroused, and is then intelligent and coherent in his ideas.

Accustomed as we are to regard diarrhœa and dysentery as curable and rarely fatal diseases in temperate climates, it may be a very natural source of surprise to many on finding that they have occupied the chief place among the causes of mortality in the Crimean expedition. This feeling will give way before clearer ideas of the pathological changes induced when these diseases are aggravated by the circumstances of the services in which our forces are engaged.

The above cases are interesting, as being types of the diseases that have undermined our strength, and have even endangered the stability of our position at a most momentous period, and, therefore, any attempt to define the circumstances that have contributed to the existence and wide development of those diseases may be attended with utility.

Reviewing the term of six months, now expired, since we left the shores of Bulgaria, there is seen to have existed, throughout a winter campaign, an almost exclusive disposition to diseases affecting the organs of digestion and assimilation. This of itself would point to morbific causes of a nature wholly distinct from those which ordinarily determine the relative frequency of diseases, because, under common circumstances of winter season in a climate rather more temperate than that of the British isles, the maladies that affect the glandular system, the respiratory organs, and those of locomotion, would be expected to prevail. In estimating the causes of this diversity, it should be recollected that when the army left the plains of Bulgaria to combat on the heights of the Crimea, the cholera was not extinct from its ranks; that during the passage by sea it gave ample proof of not having forsaken us, and that, after landing, it burst out with almost epidemic violence immediately after the battle of the Alma, when our troops encamped on the ground from which the enemy had been routed. It afterwards kept pace in the march by the Belbek across the valley of the Tchernayah, and settled down in the new encampments on the heights of Sebastopol and Balaclava, and then it showed itself with some intensity in ships previously exempt that were now brought within this new focus. With the advance of winter the disease declined; but its expiring embers were again fanned, at intervals, into mischievous activity, more especially so after the operations of the depressing influences engendered by the losses in the gale of the 14th November, and maintained by the subsequent continuance of wet tempestuous weather, with its consequent deficiency of supplies.

Before Christmas the cholera influences had become so reduced in intensity, that perfectly developed cases of it were of rare occurrence. Although many terminated fatally in December, yet their features differed materially from those of an earlier period. Serous diarrhœa, with mild collapse, terminating fatally by the exhaustive effects of persistent diarrhœa, after slight reaction had been set up, were the characteristics of the latest cases that bore the name of "cholera".

When the choleraic type of disease was on the wane, diarrhœa and dysentery, often complicated by jaundice from deficient excretion of bile, became very frequent complaints. These were, in October and November, as far as I can speak from cases under my treatment, manageable forms of disease, because occurring in men lately landed from their ships in vigorous health. These diseases had a certain acute and sthenic action.

As soon, however, as the causes that are known to determine the scorbutic cachexy had sufficiently lowered the general stamina of our men—vitiating the blood, depraving the secretions, and lessening the tenacity of the tissues—then changes were effected in the general expression of the bowel diseases, indicating a disposition to run rapidly into lesions of the mucous surface. At this time the patients had a care-worn countenance, a leucophlegmatic state of the vascular system, œdema of the legs and sometimes of the face, inability to exertion, from rigidity of the ham-string muscles, and, very generally, a reddened swollen state of the gums. There was more or less of blood, by exudation, in the stools, and the rectum was peculiarly liable to "prolapsus". The digestive functions were greatly interfered with; unchanged food was commonly seen in the evacuations: indeed, a case of lientery—a rare disease in the adult—occurring at this time, terminated fatally under my observation and treatment.

Bowel diseases of asthenic type are considered an "epidemic" of armies, so generally do they display themselves among men engaged in military operations; but I think they may, with equal propriety, be termed "an endemic of camps", as it rarely happens that armies are long fixed in one position without their suffering badly from them. The overcrowding of men in tents at nights, the little attention devoted to burying animal remains and removing excrementitious matter from the vicinity of the tents, the difficulty of procuring a sufficient supply of water for ablutionary as well as culinary purposes, unless encamped near a river, are circumstances which lead to habitual neglect of the secretory condition of the skin, and often leave no alternative but that of drinking impure water.

Such causes are efficient in producing adynamic fevers and inflammations of low type, and diseases of those internal organs towards which the blood is repelled from the surface with morbid impulse. They are also greatly increased in activity by the supervention of other causes which are inseparable from winter campaigns, such as constant exposure to wet and lowered temperature, the remaining long in constrained positions in the trenches and batteries while thus exposed, and the inadequate protection from an insufficiency of clothing.

When to these ordinary causes of disease are added others that produce the scorbutic diathesis, especially unvaried, and therefore imperfectly nutritious and insufficient food, which keeps up irritation of the mucous surface and its glandular structures, in addition to inducing the scorbutic cachexy ; it is then that the ordinary forms of diseases of the large intestine are aggravated into the disease known as scorbutic or pustular dysentery in professional, and as "camp dysentery" in popular terms.

The first case I have related will fall under this species of disease ; its course and its morbid changes, all tend to show its dependence on this series of morbific courses ; but the other case which was contemporaneous with it was so perfectly distinct in its phenomena, that I find difficulty in assuming that it was in any manner dependent on the same conditions.

Perhaps of the fatal bowel diseases of this winter, as many have belonged to the one variety as to the other. But whether the diarrhœal affection attended with softening of the villous surface, is a continuous link with the last cases of cholera recognised by that name, and has arisen, in reality, from lingering choleraic influences, I have not data sufficient to determine ; from what I have seen, I am inclined to entertain that opinion.

*H.M.S. Diamond, Balaclava.*
*10th March* 1855.

